# Quantitative Methods for Analyzing Intimate Partner Violence in Microblogs: Observational Study

**DOI:** 10.2196/15347

**Published:** 2020-11-19

**Authors:** Christopher Michael Homan, J Nicolas Schrading, Raymond W Ptucha, Catherine Cerulli, Cecilia Ovesdotter Alm

**Affiliations:** 1 Rochester Institute of Technology Rochester, NY United States; 2 University of Rochester Medical Center Rochester, NY United States

**Keywords:** intimate partner violence, social media, natural language processing

## Abstract

**Background:**

Social media is a rich, virtually untapped source of data on the dynamics of intimate partner violence, one that is both global in scale and intimate in detail.

**Objective:**

The aim of this study is to use machine learning and other computational methods to analyze social media data for the reasons victims give for staying in or leaving abusive relationships.

**Methods:**

Human annotation, part-of-speech tagging, and machine learning predictive models, including support vector machines, were used on a Twitter data set of 8767 #WhyIStayed and #WhyILeft tweets each.

**Results:**

Our methods explored whether we can analyze micronarratives that include details about victims, abusers, and other stakeholders, the actions that constitute abuse, and how the stakeholders respond.

**Conclusions:**

Our findings are consistent across various machine learning methods, which correspond to observations in the clinical literature, and affirm the relevance of natural language processing and machine learning for exploring issues of societal importance in social media.

## Introduction

Intimate partner violence (IPV) encompasses physical violence, sexual violence, stalking, and psychological aggression (including coercive acts) by a current or former intimate partner [1]. The mental and physical consequences of IPV include depression, posttraumatic stress disorder, and suicidal thoughts and behaviors [2]. Physical consequences can include myriad acute and chronic health conditions, including but not limited to functional health status, cardiac health, complicated sleep histories, and higher reports of chronic pain [1,3-5]. These effects are long-lasting [1], and IPV affects people regardless of their sexual orientation or gender identity [6]. How IPV impacts any particular individual may depend on their childhood or adolescent experiences [7] or socioeconomic class [8]. For instance, women tend to be injured more severely and are killed more frequently than their male counterparts [8]. In the United States, 6.4% of men and 6.6% of women are affected by physical violence, sexual violence, or stalking annually [8]. Despite the similarity in frequencies between the genders, there are differences regarding severity and mortality, with more women reporting severe injury and dying as a result of IPV-related deaths.

A major gap in knowledge on the prevalence of IPV exists because population-level data are difficult to collect, particularly from victims [9]. Consequently, theories about why people become involved and remain in abusive relationships are based primarily on qualitative studies and surveys with small samples, or larger samples of individuals who are often in the process of help-seeking. We know less about why people leave abusive partners because the process is often out of the vision of traditional service-providing agencies.

An alternate source of quantifiable data, such as Facebook, Twitter, or Instagram, is an alternate source of quantifiable data. It provides textual narratives at a level of personal detail reminiscent of focus groups or one-on-one interviews, but over populations larger than nearly any survey. Such an unsurpassed combination of scale and detail promises great rewards to the social, behavioral, and health sciences. Although these narrative tweets are short in length, they are a potentially rich source of information about IPV. Moreover, microblogging platforms such as Twitter offer a potential venue for public health prevention messaging that can be accessed broadly.

The amount of data present in social media is too great for human IPV experts to inspect manually. However, its semistructured, qualitative nature, along with the informality of the language used, means that human judgment is needed to make sense of the data. Machine learning has been used in many other settings to bridge this gap, and it can perform certain specialized tasks nearly as well as humans [10].

In this study, we present a machine learning workflow for social media data to hear, on a national, population-level scale, from victims themselves on why they stayed with abusive partners, or what helped them to leave. As a case study, we use this workflow to perform a comparative study on Twitter data tagged by #WhyIStayed or #WhyILeft. Such hashtags provide prelabeled data for machine learning.

Given the opportunistic nature of hashtagging, it is helpful to understand the history of these particular tags. On February 15, 2014 Ray Rice, a famous professional athlete in the National Football League, was arrested to assault his then fiancée (now wife), Janay Rice (née Palmer) [11]. First, news and sports media downplayed the incident. However, on February 19th, a video of the incident surfaced that showed Rice assaulting his fiancé in an elevator and eventually dragging her limp body out by her shoulders [11]. This caused an uproar in the advocacy and sports communities, and a backlash against the victim [11] (the backlash escalated after they married, one day after he was indicted by a grand jury). In response, on September 8, 2014 activist Beverly Gooden began using the hashtag #WhyIStayed to encourage victims of abuse to tell their stories about what kept them in abusive relationships; #WhyILeft appeared soon thereafter [12]. Tweets carrying these hashtags soon numbered in the tens of thousands, as people around the world decided to share their stories of IPV.

**Figure 1:**
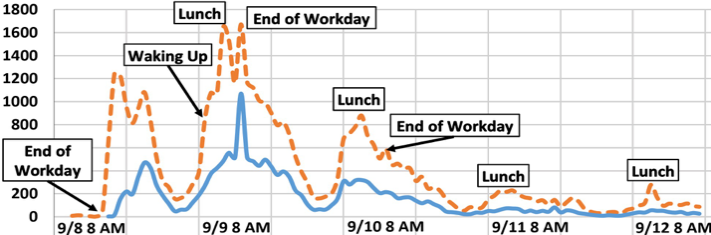
Counts per hour of #WhyIStayed (dotted) or #WhyILeft (solid) tweets from 9/8 to 9/12. Times in Eastern Standard Time, vertical lines mark 12-hour periods, with label corresponding to its left line. We removed spam from this set, but not meta tweets.

As an organizing principle and example of how to make sense out of the kind of opportunities for understanding and sensemaking that such movements provide, we focus on the specific question: *Why did both #WhyIStayed and #WhyILeft go viral?* Certainly, tweets disclosing stories about staying or leaving could be framed in terms of either hashtag, and adopting one or the other alone might have benefited the movement with a single (and thus easier-to-recall) catchphrase. These hashtags were invented by victims of IPV and virally adopted by a large community of victims over a relatively brief period of time. See Figure 1. Thus, they represent, among other things, victims of IPV not just telling their stories, *but doing so in their own terms,* rather than in those of health providers, researchers, or the criminal justice system. In a seminal work exploring 32 in-depth interviews with victims, Lempert [13] suggests that telling is a significant step in seeking help because it makes *public* their *fictions of intimacy* (citing [14]). Our primary goal was to assess how information gleaned through Twitter data could inform our understanding of survivors’ lived experiences with staying in, or leaving, abusive relationships.

## Methods

### Data

We collected a corpus of tweets containing the hashtag #WhyIStayed or #WhyILeft using the Twitter and Topsy application programming interfaces, of which the latter is currently defunct. This corpus spans the beginning of September (the start of the trend) to the beginning of October 2014, when volume dropped to background levels. The majority of tweets are from the first week of the trend’s creation (Figure 1).

#### Preprocessing

To partially anonymize the data, we replaced all URLs with the generic token “url.” We removed spam tweets based on the usernames of prominent spammers and key spam hashtags such as #MTVEMA, #AppleWatch, and #CMWorld. Additionally, we removed tweets in which Twitter accounts for DiGiorno Pizza tweeted *#WhyIStayed You had pizza*. Therefore, we excluded tweets containing tokens *pizza* or *digiorno*. The resulting corpus contained over 57,000 unique tweets.

Many tweets in the dataset were reflections on the trend itself or contained messages of support to those who shared their stories of abuse, for example, *not usually a fan of hashtag trends, but #WhyIStayed is incredibly powerful. #NFL #RayRice*. These instances, here denoted *meta-tweets*, were often retweeted, but they rarely contained reasons for staying or leaving (which were the interests of this study), so we excluded any tweets containing the keywords *janay/ray rice, football, tweets, trend,* and *video*.

#### Extracting Hashtag Labels

Typically, tweets disclosed reasons for staying (, respectively, leaving) and were prepended or appended with the hashtags #WhyIStayed (, respectively, #WhyILeft)*. #WhyILeft because I gained the courage to love myself*. If the tweet contained only one of the target hashtags, the instance was labeled with that hashtag. For tweets marked by both hashtags, we split them into two identical instances, each distinctly labeled with one of the hashtags.

The resulting corpus comprised 24,861 #WhyIStayed and 8767 #WhyILeft labeled instances. This hashtag class imbalance may be a consequence of the origins and media portrayals of the trend (the tweet that started the trend contained only the hashtag #WhyIStayed, and media reports tended to refer to the “#WhyIStayed” phenomenon) rather than an indicator that more victims stay than leave. The first #WhyILeft tweet occurred hours after the first #WhyIStayed tweet, and never gained as much traction (Figure 1).

To normalize comparisons between the tweets associated with each of these hashtags, we randomly sampled from the #WhyIStayed tweets to obtain a balanced set of 8767 examples per class. Of the 8767, we held out 1315 (14.99%) of this balanced set as a final *test set* for our machine learning experiments, and left the remaining 7452 (85.00%) as the *devset* (all of the remaining analysis in this section used the devset).

Manual inspection of the devset tweets revealed that, in addition to telling stories of IPV, the tweets served other purposes. To gain insight into the coarse grained language of these remaining tweets, we randomly sampled 1000 of them from the devset (473/1000, 47.30% #WhyIStayed and 527/1000, 52.70% #WhyILeft) and annotated them according to the coding scheme shown in Table 1, that is, as advertisements, jokes, tweets about leaving, meta (ie, tweets discussing or reporting on the #WhyIStayed/#WhyILeft phenomenon), tweets about staying, or *other*.

**Table 1:** Summary of labels from all four annotators, A1 through A4, compared with the gold standard. Each cell indicates the number of tweets an annotator gave a label to.

Annotator			Ads		Jokes		Leave		Meta		Other		Stay		Total
**A1**															
	L^a^	3		6		356		57		67		38		527	
	S^b^	6		12		28		97		31		299		473	
**A2**															
	L	10		6		378		33		47		53		527	
	S	13		6		33		74		47		300		473	
**A3**															
	L	2		10		405		49		2		59		527	
	S	7		18		29		97		1		321		473	
**A4** ^c^															
	L	3		1		122		19		15		14		174	
	S	3		0		15		35		14		92		159	

^a^L: #Why I Left.

^b^S: #Why I Stayed.

^c^A4 annotated only the first 333 tweets.

Before commencing annotation, to help better understand the distinct roles of each of the two hashtags played, we removed all occurrences of them from the tweets, to see if annotators could infer from the rest of the language whether the tweet was about staying or leaving without having the hashtag as a cue. We then studied the differences between tweets about staying versus leaving in terms of ngram bag-of-word models as features, part-of-speech tags, and a restriction to subject-verb-object tweets only.

### Machine Learning on Linguistic Features

One way to address our main research question is to ask whether #WhyIStayed and #WhyILeft hashtags indicated distinct classes of micronarratives or were merely framing devices. To address this question, we trained naïve Bayes, linear support vector machine (SVM), and radial basis function (RBF) SVM classifiers from the scikit-learn python library [15], using the hashtags as ground truth, and various language features as input. We then use the SVMs to report the features that have the most predictive power. We also considered neural models [16,17], but it was harder to make sense of these results.

## Results

### Human Annotation

Four of the authors of this paper performed the annotation task. The overall agreement overlap was 0.77. Randolph’s free-marginal multirater kappa [18] score was 0.72. We chose this multirater kappa because it allows any distribution of class labels that annotators assign (ie, it is free-marginal), unlike Fleiss’ multirater kappa, which assumes a predetermined distribution [18]. According to the resulting annotations (Table 1), on average (over all annotators), 35.28% (1176/3333) of the instances were reasons for staying (S), 40.98% (1366/3333) were reasons for leaving (L), 13.83% (461/3333) were meta comments, 1.77% (59/3333) were jokes, 1.41% (47/3333) were ads, and 6.72% (224/3333) did not match prior categories (other).

The limited contextual information that such tweets provided sometimes made it difficult to interpret unambiguously. For instance *because i was slowly dying anyway* was marked as S by two annotators and L by the other two. The annotators disagreed on whether the victim decided to stay out of a sense of resignation or left because they felt there was nothing left to lose. (The ground truth label is #WhyILeft.) Another example of disagreement was *two years of bliss, followed by uncertainty and fear*. (This tweet’s label is #WhyIStayed.) However, our results show that most tweets contained enough information for humans to infer their original hashtag labels, with annotators correctly identifying L more frequently than S.

### Lexical Usage

Basic lexical statistics in the balanced devset *before* lowercasing, stoplisting, and lemmatizing are shown in Table 2. The top nine most frequent unigrams, bigrams, and trigrams of words in the balanced dataset *after* lowercasing, stoplisting, and lemmatizing are shown in Tables 3 and 4. In order for word and ngram counts to reflect the words representing *content* rather than the words serving grammatical *functions* or tweet *markup,* before making these counts, we stoplisted, lemmatized, and excluded *start-of-sentence* and *end-of-sentence* tokens from each tweet.

Each table reveals new insights into the #WhyIStayed/#WhyILeft movement, so we examine each in turn.

**Table 2:** Basic lexical statistics on the tokens and types in the two balanced sets. Types are unique tokens whereas hapax legomena are those tokens that only occur once in the data set.

Parameters	#WhyIStayed	#WhyILeft
Number of tokens	130,545	118,768
Number of types	7094	6269
Type:token ratio	0.054	0.053
Number of hapax legomena	3871	3340

**Table 3:** Top 9 most frequent unigrams (left) and bigrams (right) after preprocessing, with their respective frequencies in the Twitter devset.

Unigrams					Bigrams			
#WhyIStayed	Frequency, n	#WhyILeft	Frequency, n	#WhyIStayed		Frequency, n	#WhyILeft	Frequency, n
think	1061	love	930	think love		127	deserve better	298
love	971	realize	888	abusive relationship		112	finally realize	103
leave	872	want	702	feel like		95	realize deserve	80
abuse	754	leave	613	make feel		89	realize love	67
believe	578	know	594	try leave		78	want live	66
tell	550	better	570	emotional abuse		72	learn love	61
want	540	deserve	558	think deserve		67	want daughter	59
say	529	abuse	507	make believe		64	year old	56
know	518	life	497	kill leave		57	know deserve	55

**Table 4:** Top 9 most frequent trigrams after preprocessing, with their respective frequencies in the Twitter devset. The number in each cell indicates the number of times the trigram appeared in the dataset.

#WhyIStayed	Frequency, n	#WhyILeft	Frequency, n
make feel like	37	realize deserve better	56
pregnant hit url	25	know deserve better	40
stay abusive relationship	25	finally realize deserve	19
change conversation url	22	son deserve better	18
leave man yell	21	true love hurt	18
abusive relationship url	20	daughter deserve better	17
man yell url	20	want daughter think	15
say kill leave	20	want daughter grow	15
church support spousal	19	daughter grow think	15

### Unigrams

The six words appearing in both columns of the unigram table (*love*, *leave*, *abuse*, *want*, *and know*) reveal common themes shared by both hashtags (Table 3). Our bi- and tri- gram analyses (presented below) reveal differences in how these common words are used. However, from the unigram perspective, the commonality of these words suggests, in the case of *leave* and *abuse*, that both #WhyIStayed and #WhyILeft tweets are often framed in terms of a leaving event, and that both often acknowledge the abuse that happened. This is perhaps not surprising, as the tags themselves suggest looking back on past abusive relationships. *Love* and *want* reveal that strong emotional forces are associated with both hashtags. *Know* indicates that knowledge (or lack thereof) is associated with both hashtags.

Of the words found in one list only, *think* and *believe* in the #Stayed list and *realize* in the #Left list suggest a transition between staying and leaving that involves learning the truth about false beliefs. The presence of *tell* and *say* in the #Stayed list suggests that an abuser coerced, deceived, or in some other way, emotionally manipulated the narrator. *Deserve* and *life* in the #Left list express positive sentiments.

### Bigrams

*Deserve better*—the most common bigram by far—indicates that a change in one’s sense of self dignity or fairness plays a major role in leaving. Both *realize* and *deserve* occur three times in the #Left list, suggesting that increased awareness, particularly from the perspective of justice or fairness, were important forces in helping narrators leave abusive relationships (Table 3).

Two words (*love* and *deserve*) appear in both lists. Each is paired with *think* in #Stayed and *realized* in #Left, suggesting a change in awareness of what love and fair treatment really are, respectively. That *deserve* also appears with *know* for #Left suggests determination.

The presence of *emotional abuse* in the #Stayed list confirms that, to the narrators of these tweets, emotional manipulation played a role in their staying in abusive relationships.

One telling bigram pairing is *kill leave* from the #Stayed list and *want live* from the #Left list. They indicate that people both remained and left abusive relationships out of fear for the safety of self and loved ones (the latter being supported by considerations about children, such as *year old*, *want daughter*, in the #Left list).

### Trigrams

The most frequent trigrams in each list *make feel like* and *realize deserve better*, respectively, make an interesting pair. They seem to indicate the important role emotional manipulation plays in staying, and that leaving is precipitated by a realization or epiphany, perhaps sometimes ones that break the spell of emotional abuse (Table 4).

*Hit*, *yell*, and *kill* all appear in the #WhyIStayed list, suggesting that violence is an important force in keeping people in abusive relationships. Notably, *man* appears twice in this list. The trigram *church support spousal* shows the important role institutions and moral or religious values play.

*Better*, *deserve*, and *daughter* each appear four times in the #WhyILeft list. This shows that concerns about the welfare of dependents (note that *son* also appears on the list) or a desire for a better life (and that such a life is deserved) drive decisions to leave. The most common word in the #Stayed list, *url* (which appears four times), is harder to interpret; recall that, to protect privacy, we removed all URLs and replaced them with this token.

### Subject-Verb-Object Structures

Fourteen percent of the dev- and test-set tweets have a subject-verb-object (SVO) structure, in which (a) the abuser is doing something to the victim, or (b) the victim is explaining something about the abuser or self. Such SVO structures represent the largest proportion of the total number of dependency structures in this data. Thus, we focus on its exploration in both corpus analytics and automated classification. These SVO structures provide insight into the abuser-victim relationship while maintaining sentence-level structures large enough to convey or indicate syntactic relationships, which tend to be more interpretable than isolated words.

We used the following conditional model to identify the most *indicative* verbs in terms of predicting hashtag class (#WhyIStayed vs #WhyILeft), among SVO tweets. Starting at the lemmatized predicate verb in each dependency parse, whenever the predicate verb followed an abuser subject word and preceded a victim object word, we added it to a distribution conditioned on class. The abuser subject words were *he, bf, boyfriend, father, dad, husband, brother,* and *man* for a male abuser, *she, gf, girlfriend, mother, mom, wife, sister,* and *woman* for a female abuser, and finally, *pastor, abuser, offender, ex, x, lover, church,* and *they* were used as neutral references. The victim object words were *me, sister, brother, child, kid, baby, friend, her, him, man,* and *woman*. These are denoted here as *abusers onto victim* structures. Analogous methods were used to extract structures in which the victim was the subject. We then determined the most indicative verb predicates from these conditional frequency distributions using the following formula for each such predicate:



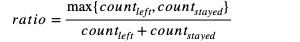



where *count_left_* and *count_stayed_* are the number of times the verb appears in #WhyILeft or #WhyIStayed tweets, respectively. Table 5 shows those where the ratio is greater than 0.70 and, to avoid a bias toward lower frequency verbs, the total count exceeds a threshold of 0.5% of the total number of instances. In the case of *abuser onto victim*, the resulting frequency threshold was 11, and in *the victim as subject*, it was 68.

**Table 5:** The most indicative (in the direction of staying) verbs for abuser onto victim and victim as subject in the tweets having subject verb object structures. An exclamation point (!) before a verb indicates negation (eg, the phrase *he did not love me* would give the verb !love). Each cell indicates the weight of each subject verb object structure, as an support vector machine feature.

Abuser onto victim	Weight of SVO^a^ structure	Victim as subject	Weight of SVO structure
convince	0.95	realize	0.98
need	0.94	think	0.91
isolate	0.94	!think	0.91
promise	0.92	find	−0.88
love	0.90	learn	−0.88
!love	−0.89	believe	0.86
!hit	0.89	!know	0.84
have	0.87	try	0.80
leave	−0.80	felt	0.73
tell	0.80	know	−0.71
be	0.78	tell	0.71
find	0.76	get	−0.70
choke	−0.75	N/A^b^	N/A
kill	−0.74	N/A	N/A

^a^SVO: subject-verb-object.

^b^N/A: not applicable.

Table 5 shows that physical *abusers on victim* verbs like *choke* and *kill* are associated with #WhyILeft, whereas for the *victim as subject* verbs *realize* appears as the most indicative verb in the data, along with *find* and *learn*. Additionally, a predominance of verbs about cognitive manipulation appear in #WhyIStayed tweets, such as *convince*, *promise*, *believe*, *think*, *!think* (where “*!*” denotes negation, for example, *I did not think he would...*) and *tell*. Heise et al [19] suggested that emotional dependence and an optimistic hope for change are reasons for staying, which these manipulative verbs seem to corroborate. Other interesting findings are the equal and opposite effects of *love* and *!love*, and the verb *!hit*, which suggests that perhaps the narrators believed they were not experiencing abuse because it was not physical, or that they feared physical retribution if they tried to leave.

### Machine Learning on Linguistic Features

We used naïve Bayes, logistic regression, linear SVM, and RBF SVM classifier methods to automatically predict whether a tweet was tagged with #WhyIStayed or #WhyILeft. The RBF SVM method performed slightly better than the others, achieving a maximum accuracy of 78% (SD 1%) on the devset and 78% on the test set using a subset of features and hyperparameters: max df=12%, C=10, gamma=1. To better understand which linguistic features and preprocessing steps were most important to these classifiers, we performed feature ablation, following the procedure in Fraser et al [20], to determine the most important features the classifier used for prediction. Interestingly, the SVO features combined with ngrams worsened performance slightly, perhaps because of trigrams capturing the majority of SVO cases, but likely also because they just covered a small fraction of the dataset. The highest accuracy, nearly 78% on the test set, used a combination of ngrams and retweet counts for features and informal register (tone) replacement in the preprocessing step.

We then used the *confidence* score of the linear SVM (defined as the distance from the classifier’s separating hyperplane in the feature space of the model) on each feature, taken as a single input to the SVM, as an estimate of that feature’s discriminativeness, or ability to distinguish between the hashtag class labels (Tables 5 and 6). This method can be seen as an alternative to the ngram count, which measures the predictive power of each ngram, rather than its frequency [21].

**Table 6:** Top 10 features, with their linear support vector machine weights using ngrams and retweet counts as features, and informal register replacement during preprocessing. Except for *try leave*, the top features were all unigrams.

#WhyIStayed	SVM^a^ weight	#WhyILeft	SVM weight
think	3.0	realize	3.3
believe	1.6	finally	2.4
convince	1.6	tired	1.7
tell	1.5	realise	1.4
say	1.3	daughter	1.4
try leave	1.1	son	1.4
money	1.0	die	1.3
abuser	0.9	strong	1.3
feel	0.9	kill	1.2
young	0.9	anymore	1.2

^a^SVM: support vector machine.

The SVM picked up on many of the same reasons for leaving and staying as those shown in Tables 3 and 4, but also revealed new ones, including *tired*, *finally*, and *strong*, which appear on the #WhyILeft list (Table 6). These seem to suggest less an epiphany or triggering crisis and more a sense that the narrator was aware of and tolerated abuse for a long time until it became too much to bear.

For staying, language about cognitive and verbal manipulation was prominent (*think*, *believe*, *convince*, *tell*, *say*, and *feel*). Several new reasons also appeared: *try leave*, *money*, and *young*. The phrase *try leave* backs up claims in clinical literature that it is often difficult to gain external support to leave, and that victims of abuse frequently go through cycles of abuse that involve leaving and returning multiple times [19]. Financial distress is often a key factor for staying [19,22], so it is no surprise that *money* appears as a top feature for the SVM. The word *young* suggests that many were too young to leave or too naïve (due to their youth) to recognize that their relationship was abusive.

### Subject-Verb-Object Structures

Restricting the dev- and test-sets to just those instances having an SVO structure, we trained the naïve Bayes, linear SVM, and RBF SVM. The linear SVM performed best, yielding 72% accuracy.

Table 7 shows the top SVO structures using the *confidence* score of the linear SVM on each data item. Some interesting structures not found in Table 5 appear here. For example, the #WhyILeft list reveals interventions from nonabusers (*sister tell me*). Taking a closer look at the supporting tweets, for example *because my sorority sisters and roommates told me nothing about how he treated me was okay*, suggests that these SVO structures refer to social support to which the victim has access. In the #WhyIStayed class, *church tell me* once again shows that religious institutions can play a role in keeping victims in abusive relationships. Several tweets indicated that their church condoned abuse as a means of avoiding embarrassment and divorce, for example *because the church told me that it was my responsibility as a godly wife to not embarrass him and just pray*.

**Table 7:** Top 10 subject-verb-object features for #WhyIStayed and #WhyILeft, with their support vector machine weights. An exclamation point (!) in front of a predicate verb indicates negation.

#WhyIStayed	SVM^a^ weights	#WhyILeft	SVM weights
he hurt me	1.1	he tell him	1.3
they !remember him	1.1	he !protect me	1.2
he need me	1.1	he !tell me	1.0
he convince me	1.1	he lie me	1.0
she convince me	1.1	he stab me	1.0
he give child	1.0	he do kid	0.9
he remind me	1.0	sister tell me	0.89
he wear me	1.0	she have baby	0.89
he !abuse kid	1.0	he strangle me	0.78
church tell me	0.99	he attack me	0.77

^a^SVM: support vector machine.

## Discussion

### Principal Findings

**Figure 2:**
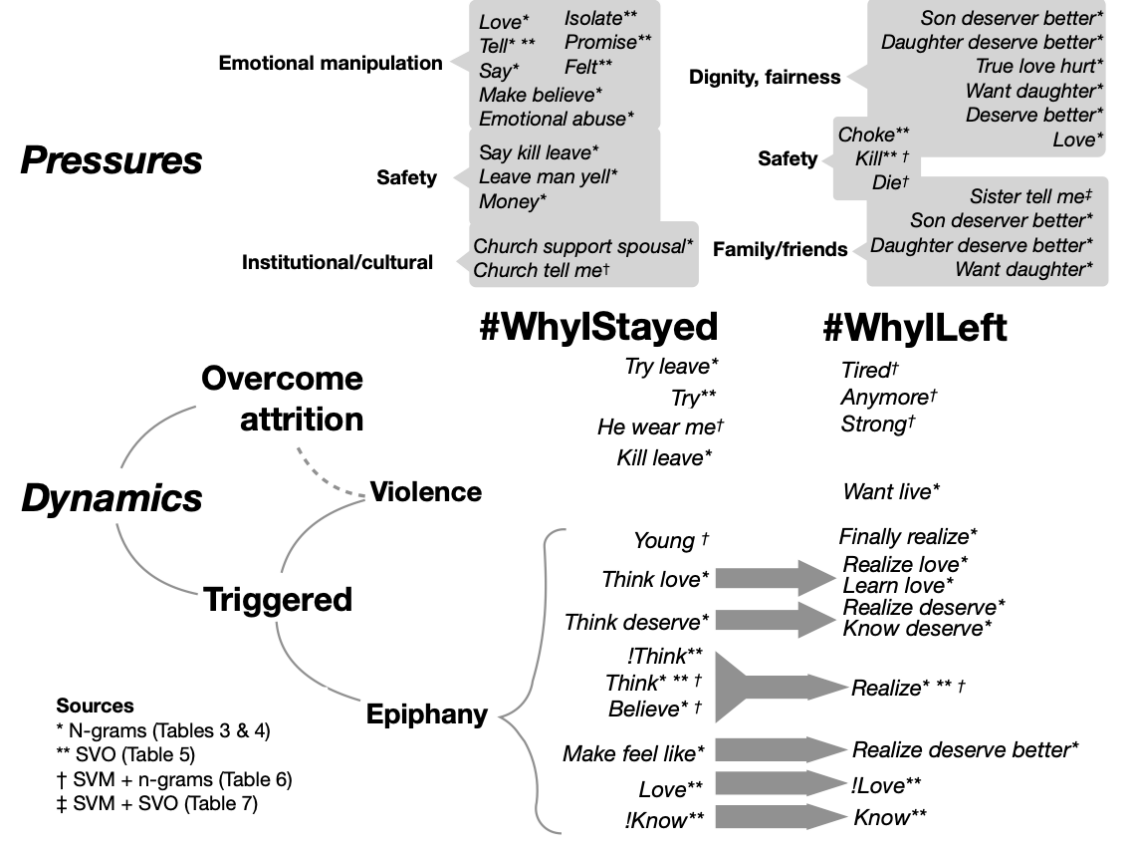
A pictorial summary of our results, grouped according to the forces that keep people in abusive relationships or cause them to leave and the dynamics involved in leaving. In the dynamics section, gray arrows denote pairs of textual features that represent opposing pressures and appeared on opposite lists in the same table (1-, 2-, or 3-gram, subject verb object, and support vector machine classification features). SVM: support vector machine; SVO: subject verb object.

Our analysis shows that the process of leaving often involves a better understanding of the reality of an abusive relationship, or coming to terms with a long anticipated but hazardous decision to leave. The words and phrases found in our results tend to be about the *pressures* of staying versus leaving or the *dynamics* involved in leaving (Figure 2). Buel [22] explains why women may choose to stay in abusive relationships, including fear of retaliation, lack of financial independence, concern for their children, emotional dependence, lack of support from friends and family, fear of divorce and the potential to lose custody of their children, and/or an optimistic hope through love that their abuser will change. Children are often a factor in keeping victims in abusive relationships, and many victims will finally leave an abusive situation once their children have grown. The words and structures found in our results support many of these observations (eg, *church tell me*, *emotional abuse*, and *daughter deserve better*).

Heise et al [19] explain why victims of abuse leave and describe the dynamics of leaving. For instance, an increase in violence sometimes triggers a realization that their abuser will not change, that things are going to get worse, that the violence is going to affect their children, that they may be killed, etc. Support from friends, family, or society often allows those abused to leave. In any case, leaving is frequently a difficult process, involving cycles of denial, self-blame, and doubt. We found many of these same pressures and dynamics in our results (eg, *realize love*, *want live*, *sister tell me*).

#### Pressures for Staying Versus Leaving

The main pressures we found for staying included emotional abuse, safety of self or other, and church communities. The pressures for leaving include a desire to be treated fairly or with dignity, the safety of self or other, and the concern for or support of a close family member.

The three pressures on each side interact with those on the other side in different ways. Emotional manipulation (a form of staying pressure) would seem to have less of an effect on victims as they become more aware of the injustice of their situation (leaving pressure). A sense of personal dignity (a leaving pressure) can cause victims to question the values of churches or other communities when they deny this dignity (a staying pressure).

For safety (both staying and leaving pressure), victims often say they stay in abusive relationships because they are afraid they will be physically or financially harmed if they leave, and they leave if they believe they or their children will be harmed or killed if they stay. Those who stay versus leave out of fear for personal safety are not necessarily the same individuals.

The last two pressures, church community and family or friends, are both about roles people outside the intimate partnership play. There is a notable asymmetry between them, in that they represent different circles in a social ecology. Several researchers have noted disparities between how institutions and victims view IPV [23,24]. That church is the only community or institution specifically mentioned in our results is not too surprising, considering the importance of beliefs and knowledge on the dynamics of leaving a relationship and the role that churches play in preserving systems of beliefs. Lempert [13] discusses the pivotal roles that peer-level families and friends can play in helping victims leave. We found some evidence for this in our results, but the most frequent relationships mentioned were victims’ children, who, as members of the family unit where the IPV occurred (and as frequent witnesses and victims themselves) were arguably in a different group from a peer-level family and friends, who were rarer in our results (Table 7).

#### Dynamics of Leaving

The dynamics of leaving can be roughly divided into two subgroups: those *triggered* by an event, such as an intervention by a friend, personal epiphany, or an imminent threat of harm, but before which the narrator was not expecting to leave, or even believed that their situation was unusually dire. The second group had to do with a slow wearing down, or *attrition,* where the victim was well aware of the harm the relationship was causing, but had to weigh those harms against the costs of leaving.

Of these groups, words and phrases related to event triggering, specifically epiphanies, seemed to be the most common. Most of these were generic, such as *thought* or *realized*. However, victims did mention that, before leaving, they failed to understand the nature of love or violence, or did not realize that the abuse they experienced was not normal. Community pressures sometimes played a role in reinforcing the feeling that the abuse was normal. Sometimes the birth or maturation of children causes the narrator to see themselves more objectively. Sometimes they even witnessed the abuse they experienced visited upon their children, and this triggered a deeper awareness of the grim reality of their situation.

Dynamics around violence typically involve the narrator at some point becoming aware, either slowly over time or by a sudden escalation that they are at imminent risk of serious harm to death if they remain in the relationship. Heise et al [19] suggested that victims of abuse leave after an increase in violence triggers a realization.

In attrition dynamics, either the cost of abuse begins to outweigh the cost of leaving, or the victim, after some period of weakness or disempowerment, is able to summon the strength to leave. Heise et al [19] suggest that women are often *not* passive victims of abuse. Instead, they actively attempt to maximize the safety of themselves and their children, while struggling in secret to navigate the (often insufficient) support structures available to them. Many victims return repeatedly to their abusers before leaving permanently [19]. In our data, direct evidence of this dynamic was scarce in the ngram analysis, but abundant in the SVM analysis (Tables 6 and 7). However, the fear of personal harm from leaving due to violence, impoverishment, etc. was a significant pressure in many cases, evidence of which was abundant in the ngram analysis (Tables 3 and 4).

### Ecological Model

Many of the findings from this study support a four-level ecological model [25,26] proposed by Heise et al [19] and expanded on by the World Health Organization [27]. All four levels that increase the likelihood that a man will abuse his partner are found in these data to varying degrees.

#### Individual

Ngrams like *hit* and *choke* (acceptance of violence as a means of solving issues), *childhood* (experiencing or witnessing abuse as a child)*,* and *want daughter, son deserve better* (trying to prevent their children from experiencing or witnessing abuse).

#### Relationship

Ngrams like *money* and *financial* (control of finances, economic stress) and the *abuser onto victim* verb *!love* (marital conflict)

#### Community

Ngrams like *try leave* and the *abuser onto victim* verb *isolate* (women’s isolation), *church support spousal*, and *church tell me* (social groups that condone abuse).

#### Societal

Evidence for this last level was scarce; however, the SVO structure *he need me* suggests that abusers sometimes act out of frustration with societal norms or expectations.

#### Why Did Both #WhyIIStayed and #WhyILeft Go Viral?

Our results fall short of spelling out exactly why both the #WhyIStayed and #WhyILeft tags went viral, but they do paint a rich picture of the pressures and dynamics involved in staying versus leaving abusive relationships. Certainly, many reasonable explanations for the virality of these hashtags may have nothing to do with the actual stories of abuse disclosed. Perhaps in reaction to #WhyIStayed (which was tweeted first) activists adopted #WhyILeft to send a message that was more upbeat and empowering than #WhyIStayed. Certainly, the shift from emotional abuse to self-dignity and fairness comes with a shift from passivity to a more active and empowered role. One fairly clear, consistent pattern that we observed in our results was that leaving involved significant changes in life circumstances. Perhaps it is difficult even to recall the frame of mind one was in on either side of the leaving event without some kind of framing device like a hashtag, or perhaps having one hashtag for each side of this transition emphasizes the importance and significance of the transition itself in a way that having only one hashtag in the public sphere does not.

### Limitations

We note several limitations in this research.

#### Bias Toward Female Victims and Male Abusers

We had hoped to study gender as a discriminative factor; however, instances with certain female abusers were rare (approximately 230 instances). Although it was difficult to determine a certain number, it appeared that the vast majority of the victims were female. This may be in part because males have significant inhibitions in reporting their abuse [8] and may therefore be less likely to tweet about their experiences and make their narratives public. It could also be that men do not face the same obstacles to leaving an abusive relationship, for instance, due to access to finances and/or alternative housing. Furthermore, we had no ability to stratify the data by sexual orientation, which could have implications for staying or leaving an abusive relationship for those that identify as lesbian, gay, transgender, or bisexual. There is a need for more research in this area.

#### Unique and/or Rare Forms of Abuse Missing

The properties of abuse and reasons for staying and leaving discovered in these data are affected by their relative frequency of occurrence. Unique and/or rare reasons for staying and leaving, and rare aspects of abusive relationships, may not be discovered using the methods presented here.

#### Noise

As with most social media data, it is important to know that these datasets likely contain posts by spam bots, lies by the users, or jokes that were missed by filters.

#### Handcrafted Pronouns and Lexical Items

The pronouns and lexical items used to convert the SVO features to *abuser onto victim* structures were handcrafted, potentially restricting the discriminative verbs that appear in the *Results* section.

#### Preprocessing

Lowercasing, stoplisting, and lemmatizing help to reduce dimensionality and thus improve learning performance, but case, tense, and certain ngrams that appear in the stoplist may be important features that were missed due to these preprocessing steps.

#### Length of Text

Although we recognize that these narratives are brief, qualitative methods such as free listing [28] often make use of brief texts. The notion of using words to create conceptual frameworks is not uncommon in mixed-method research. We suggest that our findings are worthy of continued, future exploration.

More broadly, our results show that when social media presents a large amount of data on a subject like IPV, even simple statistics such as ngrams can reveal a great deal of information about the nature of the subject. Machine learning methods, with their biases toward specific decision-making outcomes, reveal different insights. Although none of these approaches yielded effective predictive models, they provided data that were qualitative and quantitative enough to lend support to existing theories of IPV.

### Comparison With Prior Work

Computational methods are applied to better understand the #WhyIStayed/#WhyILeft movement. Recent studies of this movement are concurrent with ours [29,30]. However, they were about their historical and qualitative aspects, or were based on a tiny sample of the data available. Our work is complementary to these studies, and our goal was to provide quantitative results that lend insight and credibility to these prior qualitative and clinical observations.

An extensive body of work explores how to extract affective information and other subjective signals from social media [31-35]. Adding part of speech tags to ngrams is often attempted as well as creating word classes via data inspection, using morphosyntactic features, and exploiting the sentiment of text instances. For instance, in Xu et al [36], linear models with ngrams are recommended for their simplicity and high accuracy, although in Lamb et al [37], word classes, Twitter-specific stylometry (retweet counts, hashtags, user mentions, and emoticons), and an indicator for phrases beginning with a verb were found to be helpful over ngrams on two different tasks.

Many of these works are motivated by commercial applications, for example, mining to extract individuals’ sentiments about products or services [38,39]. Another stream of research focuses specifically on modeling, extracting, and/or tracking emotions on social media. Some of these works deal with emotions independent of context [40-42]. Other studies have studied their correlations with time [43,44] or other socioeconomic phenomena [45]. Still others model emotion as a social contagion [46-48] or focus on specific contexts, such as employment [49]. More recently, researchers have focused on specific emotional conditions or behavioral phenomena.

With respect to behavioral health, Coppersmith et al [50-52] built classifiers for detecting a number of mental health conditions, including major depression, posttraumatic stress disorder, seasonal affective disorder, and attention deficit hyperactivity disorder, by training tweets that match regular expressions related to each condition. De Choudhury et al [53,54] collected labels to consider as ground truth regarding the presence of major depression using crowd sourcing. Other researchers have focused on specific health issues, including posttraumatic stress disorder [51], early detection of epidemics [37,55], and bullying tweets [36,56]. A number of recent papers have studied suicidality on social media [57,58], or risk factors for suicide such as distress [59]. Closer to this paper and concurrent with our research, sexual abuse disclosures via anonymous Reddit were studied qualitatively by O’Neill [33] and quantitatively by Andalibi et al [60]. Subramani et al [61] performed a similar quantitative analysis of Facebook pages related to domestic violence. Karlekar and Bansal [10] studied the use of machine learning to extract narratives of sexual harassment from the SafeCity web-based forum.

### Conclusions

The research presented here demonstrates the power of social media to uncover meaningful structural, semantic, linguistic, and textual characteristics, including actions, stakeholders, and situations related to abusive relationships. It revealed micronarratives in tweeted reasons for staying versus leaving abusive relationships. A classifier for distinguishing between tweeted reasons for staying versus leaving abusive relationships achieved an accuracy of 78%. Our textual analysis, in showing that partners leave violent relationships after an epiphany of self-realization, is validated in the clinical literature. Moreover, the sheer volume of data present in social media suggests the potential to learn more details about the nature and dynamics of interpersonal violence than—due to the stigma and shame related to disclosing stories of victimization that the #WhyIStayed/#WhyILeft movement has helped erode—are currently known and may potentially help clinicians to reduce the harm caused by abusive relationships.

There are a number of interesting directions for future work. For instance, social media data with course-grained geotags could be used to study whether reasons for staying and leaving differ across geographical locations, or how varying community-level characteristics of those locations (eg, poverty level, population density, education levels, etc.) affect IPV victims. Analysis of web abuse discourse across varied media would strengthen the present findings if they overlapped, and perhaps lead to a better understanding of how victims make sense of and manage IPV and abuse.

## Abbreviations

IPV: intimate partner violence
RBF: radial basis function
SVM: support vector machine
SVO: subject-verb-object
